# Immunoglobulin, nucleos(t)ide analogues and hepatitis B virus recurrence after liver transplant: A meta‐analysis

**DOI:** 10.1111/eci.13575

**Published:** 2021-05-03

**Authors:** Quirino Lai, Gianluca Mennini, Francesco Giovanardi, Massimo Rossi, Edoardo G. Giannini

**Affiliations:** ^1^ General Surgery and Organ Transplantation Unit Department of General and Specialistic Surgery Umberto I Polyclinic of Rome Sapienza University of Rome Rome Italy; ^2^ Gastroenterology Unit Department of Internal Medicine University of Genoa IRCCS Ospedale Policlinico San Martino Genoa Italy

**Keywords:** adefovir, entecavir, lamivudine, liver transplantation, nucleos(t)ide analogues, prophylaxis

## Abstract

**Background:**

Prophylaxis with hepatitis B immunoglobulin (HBIG) represents an efficient strategy for reducing the risk of hepatitis B virus (HBV) recurrence after liver transplantation (LT). Unfortunately, the long‐term use of HBIG presents high costs. Therefore, the use of prophylaxis based only on nucleos(t)ide analogues (NUC) has been recently postulated. The present meta‐analysis aimed to evaluate the impact of HBIG ± NUC vs HBIG alone or NUC alone in post‐LT HBV recurrence prophylaxis.

**Materials and methods:**

A systematic literature search was performed using PubMed and Cochrane databases. The primary outcome investigated was the HBV recurrence after LT. Three analyses were done comparing the effect of (a) HBIG + NUC vs HBIG alone; (b) HBIG+NUC vs NUC alone; and (c) HBIG alone vs NUC alone. Sub‐analyses were also performed investigating the effect of low and high genetic barrierto‐recurrence NUC.

**Results:**

Fifty‐one studies were included. The summary OR (95%CI) showed a decreased risk with the combination of HBIG + NUC vs HBIG alone for HBV recurrence, being 0.36 (95% CI = 0.22‐0.61; *P* < .001). HBIG + NUC combined treatment reduced HBV reappearance respect to NUC alone (OR = 0.22; 95% CI = 0.16‐0.30; *P* < .0001). Similarly, HBIG alone was significantly better than NUC alone in preventing HBV recurrence (OR = 0.20; 95% CI = 0.09‐0.44; *P* < .0001).

**Conclusions:**

Prophylaxis with HBIG is relevant in preventing post‐LT HBV recurrence. Its combination with NUC gives the best results in terms of protection. The present results should be considered in light of the fact that also old studies based on lamivudine use were included. Studies exploring in detail high genetic barrier‐to‐recurrence NUC and protocols with definite use of HBIG are needed.

## 1 INTRODUCTION

Hepatitis B virus (HBV) represents a major global health problem worldwide.^1^ According to the World Health Organization estimations, approximately 300 million people have been infected with chronic HBV, with two‐thirds being in Asia.^2^ HBV‐related end‐stage liver disease (ie acute liver failure and cirrhosis) and its complication hepatocellular carcinoma are among the principal indications for liver transplantation (LT).^3^ However, transplanted patients without any prophylaxis may suffer from HBV recurrence in up to 80% of cases.^4^ Hepatitis B immunoglobulin (HBIG) represents an efficient passive immune agent against HBV, and long‐term passive immunoprophylaxis after LT results in a 60%‐80% reduction of HBV recurrence.^5^ Unfortunately, long‐term HBIG usage presents some drawbacks, such as relevant costs and the need to repeatedly monitor hepatitis B surface antibody levels.^6^

In the clinical practice, following the introduction of the nucleoside analogue lamivudine (LAM) combined with HBIG, a further reduction of the HBV recurrence rates has been reported.^7^ However, LAM has a low genetic barrier‐to‐resistance.^8^ Currently, more potent drugs with a high genetic barrier‐to‐resistance—such as the nucleos(t)ide analogues (NUC) adefovir (ADV), entecavir (ETV) and tenofovir (TDF)—have been introduced to avoid the risk of viral recurrence in transplanted patients.^9^, ^10^

Due to their potent effect, the exclusive prophylactic use of high genetic barrier‐to‐resistance NUC without HBIG has been proposed to avoid the problems associated with long‐term immunoprophylaxis.^11^ With the intent to gain a better insight into this issue, a meta‐analysis has been performed to evaluate the practical necessity of HBIG in the prophylaxis of post‐LT HBV recurrence. To this end and to explore all the potential clinical settings, the HBV recurrence rates after LT were compared in patients receiving prophylaxis based on (a) HBIG alone vs HBIG+NUC; (b) HBIG alone vs NUC alone; and (c) HBIG+NUC vs NUC alone. We further performed some sub‐analyses to investigate the role of low and high genetic barrier‐to‐resistance NUC.

## 2 MATERIALS AND METHODS

### 2.1 Search sources and study design

A systematic review of the published literature focused on the role of HBIG in the prophylaxis of HBV recurrence after LT was undertaken. The search strategy was performed following the Preferred Reporting Items for Systemic Reviews and Meta‐Analysis (PRISMA) guidelines.^12^

The specific research question formulated in the present study includes the following Patients, Intervention, Comparator, Outcome (PICO) components:

Patient: patient with end‐stage acute or chronic HBV‐related liver disease undergoing LT;

Intervention: prophylaxis based on HBIG (±NUC);

Comparison: prophylaxis based on NUC alone;

Outcome: HBV recurrence after LT, defined as the detectability of HBsAg or HBV DNA during the study period.

A search of the PubMed and Cochrane Central Register of Controlled Trials Databases was conducted using the following terms:

(HBV) AND (liver transplant*) AND (recurrence). The search period was from ‘2000/01/01’ to ‘2020/11/09’.

The systematic qualitative review included only English studies that included human patients. Published reports were excluded based on several criteria: (a) data on animal models; (b) lacked enough clinical details; and (c) had nonprimary source data (eg review articles, nonclinical studies, letters to the editor, expert opinions and conference summaries). In the case of studies originating from the same centre, the possible overlapping of clinical cases was examined, and the most informative study was considered eligible.

### 2.2 Data extraction and definitions

Following a full‐text review of the eligible studies, two independent authors (QL and EGG) performed the data extraction and crosschecked all outcomes. During selecting articles and extracting the data, potential discrepancies were resolved following a consensus with a third reviewer (GM). Collected data included the first author of the publication, year of publication, country and the number of treated and recurred cases according to the different therapies adopted.

### 2.3 Quality assessment

Selected studies were systematically reviewed with the intent to identify potential sources of bias. The papers' quality was assessed using the Risk of Bias In Non‐randomized Studies of Interventions (Robins‐I) tool.^13^

### 2.4 Statistical analysis

Study results were expressed as odds ratio (OR) with 95% confidence intervals (95% CIs). The statistical heterogeneity was evaluated with the Higgins statistic squared (I2). I2 values of 0%‐25% were considered as an index of low heterogeneity between studies, 26%‐50%: moderate heterogeneity and ≥51%: high heterogeneity. The fixed‐effects model was used when low or moderate (0%‐50%) heterogeneity was detected between studies, while the random effects model was preferred when high heterogeneity was present. The value *P* < .05 was considered indicative of statistical significance.

The meta‐analysis was performed using OpenMetaAnalyst (http://www.cebm.brown.edu/openmeta/index.html).

## 3 RESULTS

### 3.1 Search results and study characteristics

**FIGURE 1:**
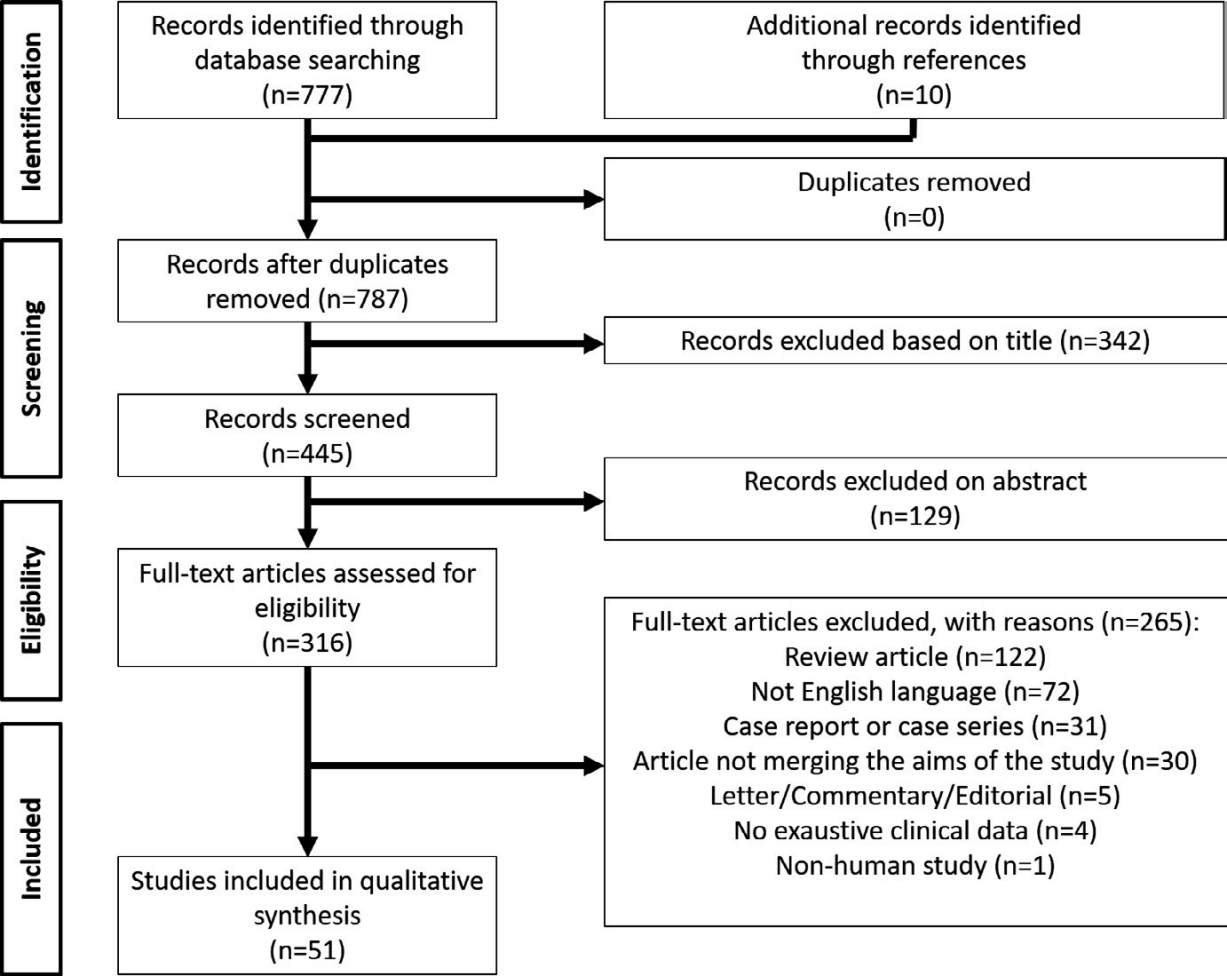
PRISMA summarizing the trial flow

The PRISMA flow diagram schematically depicts the article selection process (Figure 1). Among the 777 articles screened, a total of 51 studies were lastly included in this review.^14^, ^15^, ^16^, ^17^, ^18^, ^19^, ^20^, ^21^, ^22^, ^23^, ^24^, ^25^, ^26^, ^27^, ^28^, ^29^, ^30^, ^31^, ^32^, ^33^, ^34^, ^35^, ^36^, ^37^, ^38^, ^39^, ^40^, ^41^, ^42^, ^43^, ^44^, ^45^, ^46^, ^47^, ^48^, ^49^, ^50^, ^51^, ^52^, ^53^, ^54^, ^55^, ^56^, ^57^, ^58^, ^59^, ^60^, ^61^, ^62^, ^63^, ^64^

Thirty‐seven (72.5%) studies included in the analytic cohort were published during 1999‐2009 and the remaining 14 (27.5%) during the last decade. Twenty articles (39.2%) were from Asia, of whom 11 (21.6%) were from China, while sixteen studies (31.4%) were from Europe and 11 (21.6%) from North America. In two cases (3.9%), International studies were reported (Figure S1).

### 3.2 Qualitative assessment of the included studies

Results from the qualitative assessment of the included studies are depicted in Figure S2. Among the 51 selected papers, six (11.8%) were prospective randomized controlled trials, one (2.0%) was a prospective study without a comparison arm, and three (5.9%) were prospective studies with a historical comparison arm. Overall, ten studies (19.6%) were designed as prospective analyses, and the remaining 41 studies (80.4%) were retrospective. As for the ROBINS‐I tool quality assessment, 41 studies had a low risk of bias, while ten studies showed a high or unclear risk for bias.

### 3.3 Review of the eligible studies

Data extracted from the selected articles are reported in detail in Table 1. The only study based on a population of LT patients, including more than 1000 cases, came from Korea (n = 1524), while the sample size was above 100 cases in 13 studies.^22^, ^57^, ^58^, ^60^, ^62^, ^64^ In the remaining 37 studies, the sample size was smaller than 100 cases.

**TABLE 1:** Characteristics of included studies

Author	Year	Country	Ref	Design	Period	HBIG	N	Rec	HBIG+NUC	N	Rec	NUC	N	Rec
McCaughan	1999	Australia	^14^	Prospective^a^	1988‐97	HBIG	10	9	HBIG+LAM	9	0		/	/
Han	2000	USA	^15^	Retrospective	1991‐98	HBIG	12	3	HBIG+LAM	59	0		/	/
Seehofer	2001	Germany	^16^	Retrospective	1988‐00	HBIG	40	19	HBIG+LAM	17	3		/	/
Naoumov	2001	International	^17^	RCT	/	HBIG	12	1		/	/	LAM	12	2
Marzano	2001	Italy	^18^	Prospective^a^	1990‐96	HBIG	12	6	HBIG+LAM	26	1		/	/
Lee	2001	Korea	^19^	Retrospective	1996‐99	HBIG	19	3	HBIG+LAM	24	3		/	/
Yuan	2002	China	^23^	Retrospective	/		/	/	HBIG+LAM	2	0	LAM	13	1
Park	2002	Korea	^21^	RCT	1996‐00	HBIG	31	1		/	/	LAM	30	3
Honaker	2002	USA	^20^	Retrospective	1990‐01	HBIG	14	3	HBIG+LAM	9	0		/	/
Anselmo	2002	USA	^22^	Retrospective	1984‐01	HBIG	28	13	HBIG+LAM	89	10	LAM	20	13
Zhu	2003	China	^27^	Retrospective	/		/	/	HBIG+LAM	9	0	LAM	15	3
Roche	2003	France	^29^	Retrospective	1986‐98	HBIG	259	62	HBIG+NUC	25	2		/	/
Dumortier	2003	France	^24^	Prospective^a^	1990‐01	HBIG	43	10	HBIG+LAM	17	0		/	/
Ben‐Ari	2003	Israel	^25^	Retrospective	1992‐00	HBIG	24	6	HBIG+LAM	9	1		/	/
Buti	2003	Spain	^28^	RCT	1998‐02		/	/	HBIG+LAM	15	1	LAM	14	3
Sousa	2003	Spain	^26^	Retrospective	1990‐00	HBIG	17	4	HBIG+LAM	10	0		/	/
Xia	2004	China	^32^	Retrospective	/		/	/	HBIG+LAM	43	1	LAM	15	3
Wang	2004	China	^31^	Retrospective	2002‐03		/	/	HBIG+LAM/ADV	66	2	LAM	2	1
Neff	2004	USA	^30^	Retrospective	1994‐03		/	/	HBIG+LAM	41	5	LAM	51	9
Lo	2005	Hong Kong	^34^	Retrospective	1999‐04		/	/	HBIG+LAM+ADV	8	0	LAM/ADV	8	2
Marzano	2005	Italy	^33^	Retrospective	1990‐02	HBIG	98	9	HBIG+LAM	79	6		/	/
Zheng	2006	China	^35^	Retrospective	1999‐04		/	/	HBIG+LAM	114	16	LAM	51	21
Wu	2006	China	^36^	Retrospective	/		/	/	HBIG+LAM	114	16	LAM	75	12
Jiao	2007	China	^39^	Retrospective	1999‐05		/	/	HBIG+LAM	56	3	LAM	28	7
Caccamo	2007	Italy	^42^	Retrospective	1992‐04	HBIG	21	0	HBIG+LAM	25	0		/	/
Yi	2007	Korea	^37^	Retrospective	1999‐02	HBIG	95	6	HBIG+LAM	108	15		/	/
Buti	2007	Spain	^38^	RCT	1998‐00		/	/	HBIG+LAM	15	0	LAM	14	0
Yoshida	2007	USA	^40^	Retrospective	1994‐04		/	/	HBIG+LAM	25	3	LAM	22	3
Schiff	2007	USA	^41^	Prospective	/		/	/	HBIG+LAM+ADV	34	2	LAM+ADV	23	2
Wong	2007	USA	^43^	Retrospective	1994‐05	HBIG	6	0	HBIG+LAM	15	1		/	/
Angus	2008	International	^45^	RCT	2004‐06		/	/	HBIG+LAM	18	0	LAM+ADV	16	0
Avolio	2008	Italy	^47^	Retrospective	1988‐07	HBIG	16	3	HBIG+LAM	26	2		/	/
Freshwater	2008	UK	^46^	Retrospective	/		/	/	HBIG+LAM/LAM+ADV	24	1	LAM	10	3
Yilmaz	2008	USA	^44^	Retrospective	1985‐05	HBIG	25	8	HBIG+LAM	16	0		/	/
Dai	2009	China	^48^	Retrospective	/		/	/	HBIG+LAM	42	2	LAM	13	10
Ma	2009	China	^49^	Retrospective	/		/	/	HBIG+LAM	210	8	LAM	106	12
Beckebaum	2009	Germany	^50^	Retrospective	1992‐07	HBIG	43	2	HBIG+NUC	52	2	NUC	9	6
Pauwelyn	2010	Belgium	^51^	Retrospective	1992‐08	HBIG	29	3	HBIG+LAM/LAM+ADV	27	5		/	/
Hwang	2011	Korea	^54^	Retrospective	1992‐09	HBIG	1463	106	HBIG+NUC	61	0		/	/
Campos‐Varela	2011	Spain	^53^	Retrospective	1988‐08	HBIG	7	5	HBIG+NUC	42	3		/	/
Ahn	2011	USA	^52^	Retrospective	2002‐07	HBIG	7	0	HBIG+NUC	17	3	NUC	4	1
Yuan	2013	China	^55^	Retrospective	2000‐11		/	/	HBIG+LAM	16	1	LAM	6	3
Lee	2013	Korea	^57^	Retrospective	1996‐10	HBIG	346	55	HBIG+ETV	207	8		/	/
Teperman	2013	USA	^56^	RCT	/		/	/	HBIG+FTC/TDF	19	0	FTC/TDF	18	0
Zhang	2014	China	^58^	Retrospective	1999‐10		/	/	HBIG+LAM	156	3	LAM	28	8
Teegen	2018	Germany	^60^	Retrospective	1988‐16	HBIG	97	39	HBIG+LAM or ETV/TDF	243	53		/	/
Ajayi	2018	USA	^59^	Retrospective	2013‐16	HBIG	28	0		/	/	NUC	25	0
Darweesh	2019	Egypt	^61^	Retrospective	2008‐16		/	/	HBIG+NUC	42	5	NUC	2	0
Dobrindt	2020	Germany	^62^	Retrospective	1988‐13	HBIG	40	0	HBIG+NUC	141	0		/	/
Park	2020	Korea	^64^	Retrospective	2014‐/	HBIG	121	2	HBIG+NUC	196	3	NUC	9	1
Muthiah	2020	Singapore	^63^	Retrospective	2001‐15	HBIG	20	0	HBIG+NUC	3	1	NUC	35	6

Abbreviations: ADV, adefovir; ETV, entecavir; FTC, emtricitabine; HBIG, hepatitis B immunoglobulin; LAM, lamivudine; N, number; NUC, nucleos(t)ide analogues; RCT, randomized controlled trial; Rec, recurrence; Ref, reference; TDF, tenofovir.

^a^ Prospective arm compared with a historical group.

Of the 51 included studies, 22 compared HBIG alone vs HBIG+NUC combination therapy,^14^, ^15^, ^16^, ^29^, ^33^, ^37^, ^42^, ^43^, ^44^, ^47^, ^51^, ^53^, ^54^, ^57^, ^60^, ^62^ and 21 compared HBIG+NUC vs NUC alone.^23^, ^27^, ^38^, ^39^, ^40^, ^41^, ^45^, ^46^, ^48^, ^49^, ^55^, ^56^, ^58^, ^61^ In three studies, HBIG alone was compared with NUC alone.^17^, ^21^, ^59^ In five studies, all the three different groups were reported.^23^, ^50^, ^52^, ^63^, ^64^

### 3.4 HBIG+NUC vs HBIG alone

**FIGURE 2:**
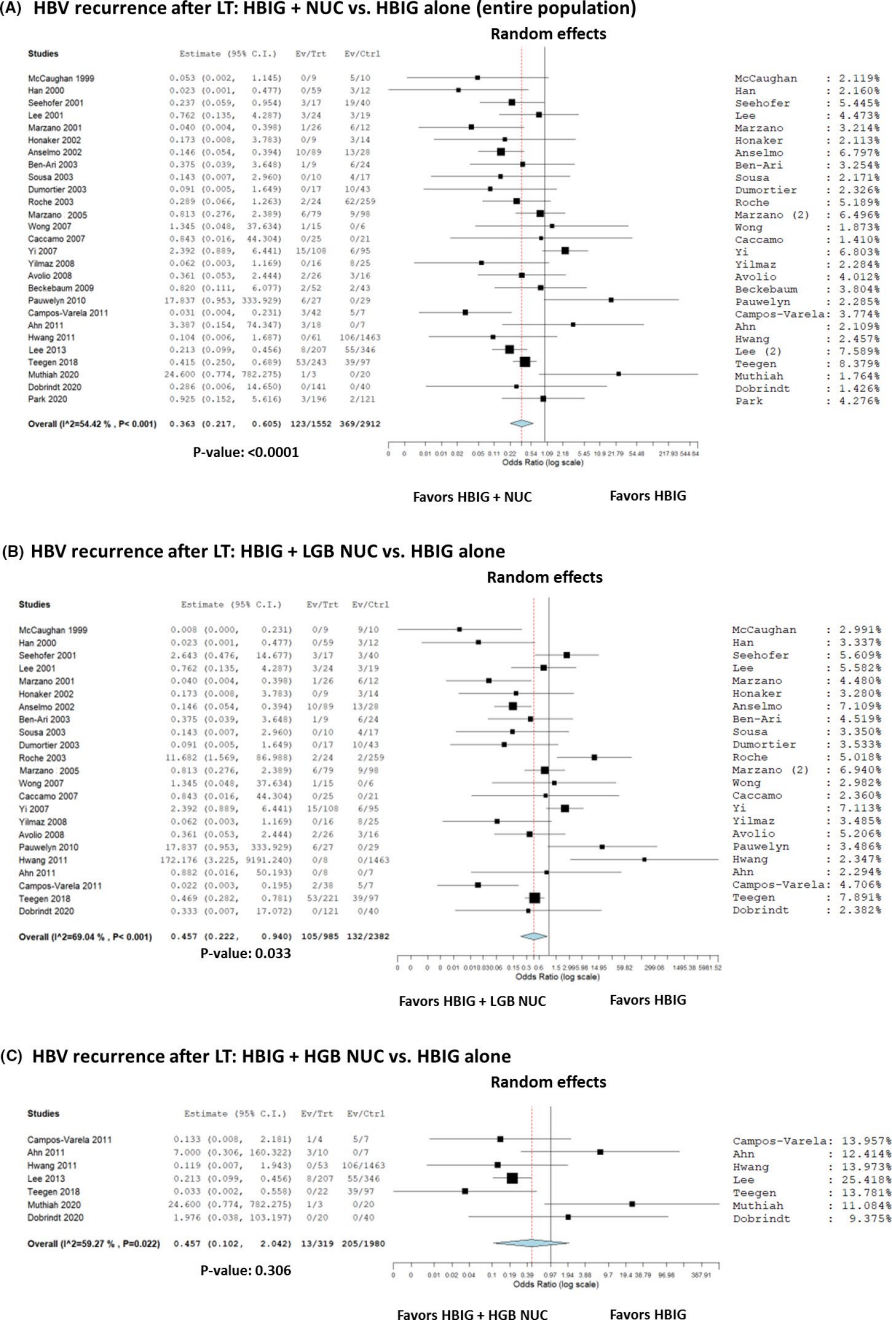
A‐C, Forest plot of odds ratios and 95% confidence intervals for the association between HBIG+NUC vs HBIG alone for the risk of HBV recurrence in patients undergoing liver transplantation. A, entire population; (B) HBIG +low genetic barrier‐to‐recurrence NUC vs HBIG alone.; (C) HBIG+high genetic barrier‐to‐recurrence NUC vs HBIG alone

According to the data shown in Table 2, 27 studies reported post‐LT HBV recurrence data in patients receiving HBIG+NUC vs HBIG alone. A total of 4464 patients were considered, with 496 (11.1%) recurrences. In detail, 123/1552 (7.9%) and 373/2912 (12.8%) recurrences were observed in the HBIG+NUC and HBIG alone group, respectively. Most of the studies showed a benefit of HBIG+NUC combination therapy over HBIG alone (Figure 2A). The summary OR (95% CI) showed a decreased risk with the combination of HBIG and NUC vs HBIG alone for HBV recurrence, being 0.36 (95% CI = 0.22‐0.61; *P* < .001).

**TABLE 2:** Results of meta‐analytic comparison between LT patients treated with an HBV prophylaxis based on (a) HBIG +NUC vs HBIG alone; (b) HBIG +NUC vs NUC alone; and (c) HBIG alone vs NUC alone

Outcome of interest	Study (n)	HBIG+NUC (n)	Rec	HBIG alone (n)	Rec	NUC alone (n)	Rec	OR (95%CI)	*P* value	Study heterogeneity		*P* value
										*df*	I2%	
(a) HBV recurrence: HBIG+NUC vs HBIG alone	27	Tot = 1552	123	2912	373	‐	‐	0.363 (0.217‐0.605)	<.001	26	54.42	<.001
	23	LGB = 985	105	2382	314	‐	‐	0.457 (0.222‐0.940)	.033	22	69.035	<.001
	7	HGB = 319	13	1980	205	‐	‐	0.457 (0.102‐2.042)	.306	6	59.266	.022
(b) HBV recurrence: HBIG+NUC vs NUC alone	27	Tot = 1455	88	‐	‐	Tot = 638	127	0.217 (0.157‐0.299)	<.001	26	NA	<.001
	18	LGB = 1034	71	‐	‐	LGB = 483	109	0.205 (0.113‐0.371)	<.001	17	54.475	.003
	3	LGB = 50	0	‐	‐	HGB = 25	1	0.229 (0.026‐2.023)	.185	2	NA	.700
	3	HGB = 16	4	‐	‐	LGB = 19	4	0.880 (0.152‐5.107)	.887	2	NA	.514
	6	HGB = 106	8	‐	‐	HGB = 73	6	0.700 (0.217‐2.259)	.550	5	NA	.553
(c) HBV recurrence: HBIG alone vs NUC alone	7	‐	‐	262	19	Tot = 119	32	0.201 (0.091‐0.442)	<.001	6	NA	.313
	5	‐	‐	98	15	LGB = 79	21	0.339 (0.134‐0.859)	.023	4	NA	.795
	2	‐	‐	27	0	HGB = 22	2	0.218 (0.027‐1.738)	.150	1	NA	.643

Abbreviations: CI, confidence intervals; *df*, degrees of freedom; HBIG, hepatitis B immunoglobulin; HGB, high genetic barrier‐to‐recurrence; *I* ^2^, Higgins statistic squared; LGB, low genetic barrier‐to‐recurrence; n, number; NA, not available; NUC, nucleos(t)ide analogues; OR, odds ratio.

Sub‐analyses showed that patients receiving HBIG+low genetic barrier‐to‐resistance NUC vs HBIG alone showed a reduced risk of recurrence in patients undergoing a combination therapy (OR = 0.46, 95% CI = 0.22‐0.94; *P* = .03; Figure 2B).

In the case of HBIG+high genetic barrier‐to‐resistance NUC vs HBIG alone, no statistical significance was reported (OR = 0.46, 95% CI = 0.10‐2.04; *P* = .31; Figure 2C).

### 3.5 HBIG+NUC vs NUC alone

**FIGURE 3:**
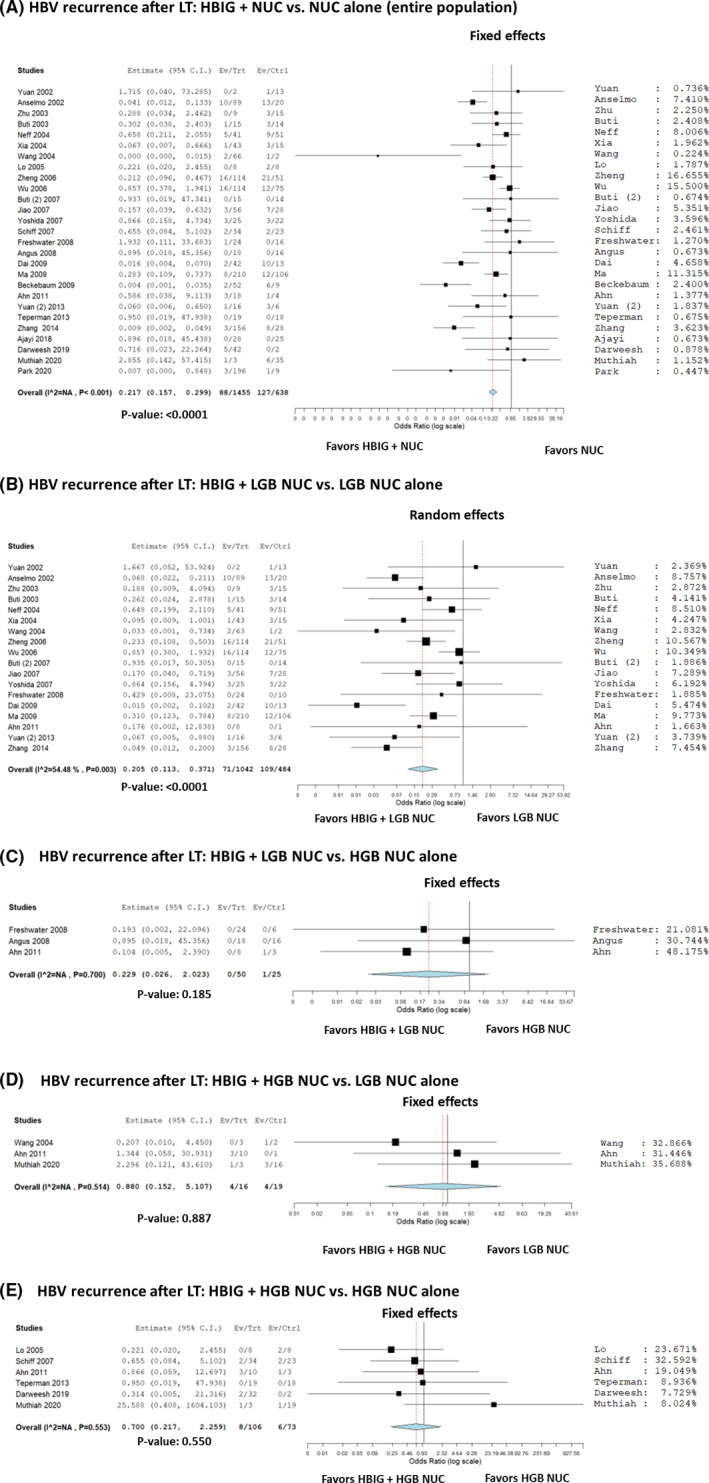
A‐E, Forest plot of odds ratios and 95% confidence intervals for the association between HBIG+NUC and NUC alone for the risk of HBV recurrence in patients undergoing liver transplantation. A, entire population; (B) HBIG+low genetic barrier‐to‐recurrence NUC vs low genetic barrier‐to‐recurrence NUC alone; (C) HBIG +low genetic barrier‐to‐recurrence NUC vs high genetic barrier‐to‐recurrence NUC alone; D, HBIG+high genetic barrier‐to‐recurrence NUC vs low genetic barrier‐to‐recurrence NUC alone; E, HBIG+high genetic barrier‐to‐recurrence NUC vs high genetic barrier‐to‐recurrence NUC alone

According to the data shown in Table 2, 27 studies reported post‐LT HBV recurrence data in patients receiving HBIG+NUC vs NUC alone. A total of 2093 patients were considered, with 215 (10.3%) recurrences. In detail, 88/1455 (6.0%) and 127/638 (19.9%) recurrences were observed in the HBIG+NUC and NUC alone group, respectively. Most of the studies showed a benefit of HBIG+NUC combination therapy over NUC alone (Figure 3A). The summary OR (95% CI) showed a reduced risk with the combination of HBIG and NUC vs NUC alone for HBV recurrence, being 0.22 (95% CI = 0.16‐0.30; *P* < .0001).

Four different sub‐analyses were performed, according to the different combinations of low and high genetic barrier‐to‐resistance NUC. When the combination therapy of HBIG+low genetic barrier‐to‐resistance NUC vs low genetic barrier‐to‐resistance NUC alone was used, a protective effect was reported (OR = 0.21, 95% CI = 0.11‐0.37; *P* < .001) (Figure 3B). All the other combinations did not show any statistically significant difference. In detail, HBIG+low genetic barrier‐to‐resistance NUC vs high genetic barrier‐to‐resistance NUC alone had an OR = 0.23 (95% CI = 0.03‐2.02; *P* = .19; Figure 3C). HBIG+high genetic barrier‐to‐resistance NUC vs low genetic barrier‐to‐resistance NUC alone had an OR = 0.88 (95% CI = 0.15‐5.11; *P* = .89) (Figure 3D). HBIG+high genetic barrier‐to‐resistance NUC vs high genetic barrier‐to‐resistance NUC alone had an OR = 0.70 (95% CI = 0.22‐2.26; *P* = .55) (Figure 3E).

### 3.6 HBIG alone vs NUC alone

**FIGURE 4:**
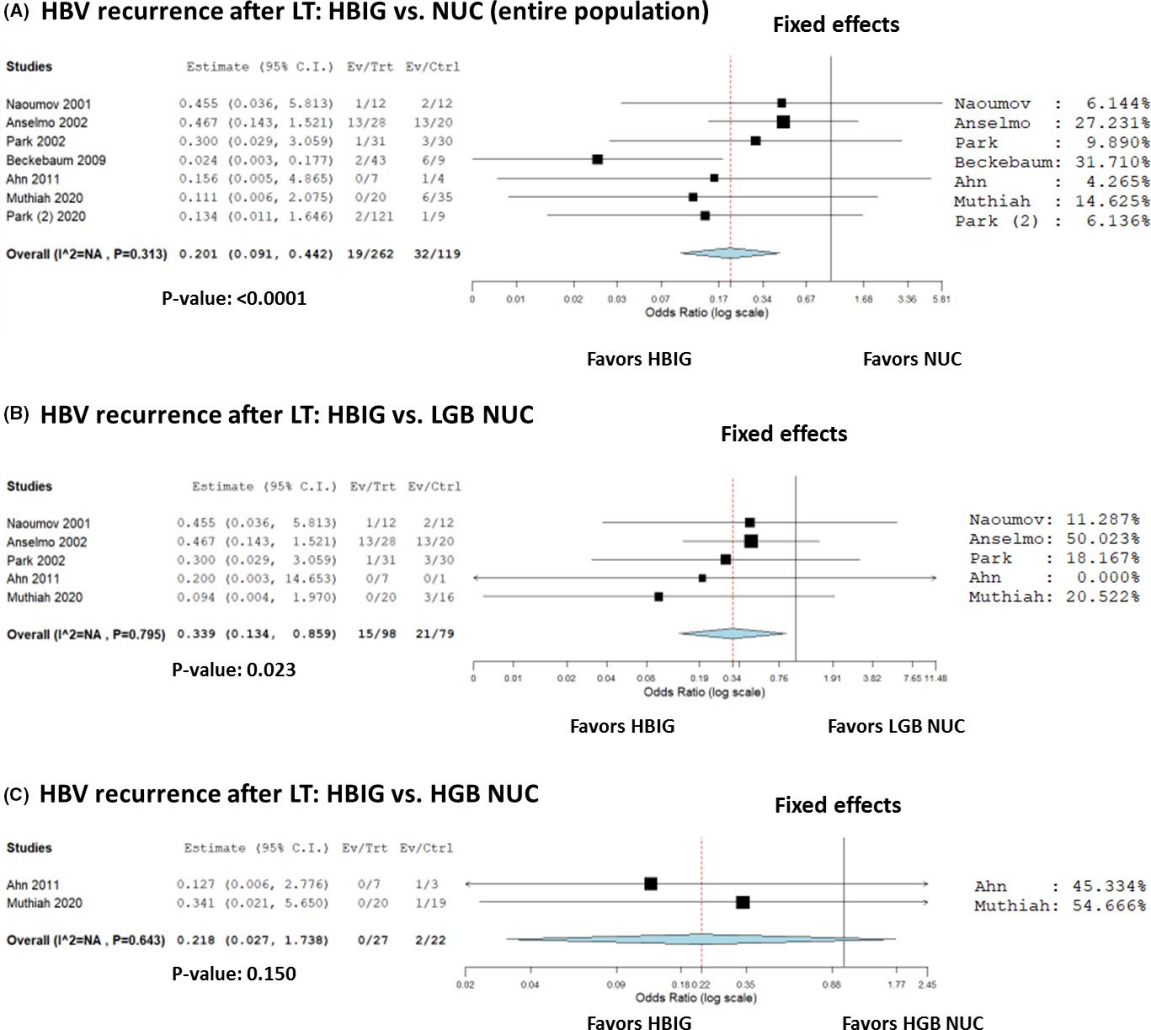
A‐C, Forest plot of odds ratios and 95% confidence intervals for the association between HBIG alone and NUC alone for the risk of HBV recurrence in patients undergoing liver transplantation. A, entire population; (B) HBIG alone vs low genetic barrier‐to‐recurrence NUC alone; (C) HBIG alone vs high genetic barrier‐to‐recurrence NUC alone

According to the data shown in Table 2, 7 studies reported post‐LT HBV recurrence data in patients receiving HBIG alone vs NUC alone. A total of 381 patients were considered, with 51 (13.4%) recurrences. In detail, 19/262 (7.3%) and 32/119 (26.9%) recurrences were observed in the HBIG alone and NUC alone group, respectively. All the studies showed a benefit of HBIG alone over NUC alone (Figure 4A). The summary OR (95% CI) showed a reduced risk with the use of HBIG alone vs NUC alone for HBV recurrence, being 0.20 (95% CI = 0.09‐0.44; *P* < .0001).

When HBIG alone vs low genetic barrier‐to‐resistance NUC alone cases were compared, a protective effect of HBIG alone was reported for the risk of HBV recurrence after LT (OR = 0.34, 95% CI = 0.13‐0.86; *P* = .02) (Figure 4B). Comparing HBIG alone vs high genetic barrier‐to‐resistance NUC alone did not show any statistically significant result (OR = 0.22, 95% CI = 0.03‐1.74; *P* = .15; Figure 4C).

## 4 DISCUSSION

**FIGURE 5:**
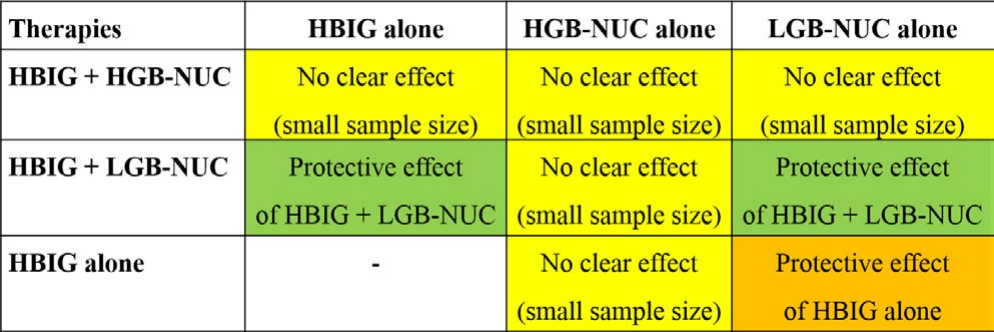
Schematic representation of the results obtained from the meta‐analyses

The results of this meta‐analysis indicated that the role of HBIG in the prophylaxis of HBV recurrence after LT is not secondary (Figure 5). When in combination therapy with NUC, the use of HBIG was markedly better than HBIG alone or NUC alone in the post‐LT setting for the prevention of HBV recurrence. Overall, using HBIG+NUC vs HBIG alone decreased the odds of HBV recurrence by 2.8‐fold. Using HBIG+NUC vs NUC alone reduced the odds of HBV recurrence by 4.6‐fold. The use of HBIG alone regimen was superior compared with NUC alone, reducing the odds of HBV recurrence by 5‐fold.

Seven meta‐analyses have already been published on the prophylaxis for preventing HBV recurrence after LT.^65^, ^66^, ^67^, ^68^, ^69^, ^70^, ^71^

In detail, Loomba R. et al (N = 317) compared HBIG+lamivudine vs HBIG alone.^65^ Rao et al^66^ (N = 551) compared LAM alone vs LAM+HBIG. Katz et al^67^ (N = 706) compared (a) HBIG alone vs combination treatment, (b) antivirals alone vs combination treatment and (c) LAM alone vs HBIG alone. Zhang et al,^68^ only including the randomized controlled trials (N = 162), compared HBIG alone, LAM alone and HBIG+LAM in a network meta‐analysis. Wang et al^69^ (N = 1484) explored the role of HBIG, also performing sub‐group analyses showing the positive impact of HBIG in patients with positive preoperative HBV DNA status. Zheng et al^70^ performed a network meta‐analysis for the risk of HBV recurrence (N = 7274) comparing the different NUC (LAM alone, ETV alone, LAM+TDF, LAM+ADV, TDF alone and ADV alone). Lastly, Li et al^71^ (N = 2374) compared (a) potent NUC+indefinite HBIG vs NUC alone and (b) NUC+finite HBIG vs NUC monotherapy.

The first consideration of the reported data is that significant heterogeneity exists in the studies published, clearly reporting the complexity of the issue and the difficulty of performing a meta‐analysis on this argument.

Interestingly, many of these studies reached conclusions similar to ours. For example, Loomba et al analysed six articles showing that HBIG+LAM reduced HBV recurrence risk compared with HBIG alone (OR = 0.08; *P* < .001).^65^ Rao et al identified six articles reporting that HBIG+LAM reduced HBV recurrence risk compared with LAM alone (relative risk = 0.38; *P* < .0001).^66^ Indeed, the advantage of the present meta‐analysis relates to the fact that the prophylactic scheme HBIG+NUC is, for the first time, contextually compared with the corresponding monotherapies (ie HBIG alone or NUC alone). Moreover, an innovative sub‐analysis was done investigating the role of low and high genetic barrier‐to‐resistance NUCs separately. Such an analysis was done with the primary intent to demonstrate that the new‐generation NUC present superiority with respect to the use of an old drug like the LAM.

Another critical element to address is the number of selected articles (N = 51) and patients (N = 6278) in the meta‐analysis. Only Zheng et al^70^ enrolled more cases; however, Zheng et al performed a network meta‐analysis, in which it is not necessary to find papers presenting control groups. Compared with other conventional meta‐analyses, our study enrolled the most significant population ever exploring this critical issue.

We feel that the results obtained in our analyses have important clinical and public policy implications, showing that the use of HBIG in combination with NUC should be preferred due to its greater efficacy in preventing HBV recurrence following LT than using HBIG or NUC alone.

The reason for the synergistic activity of HBIG+NUC should be that HBIG and antivirals prevent the recurrence of hepatitis B by different mechanisms. HBIG neutralizes circulating virus particles and induces lysis of infected hepatocytes, while antivirals directly reduce viral load in the liver and extrahepatic sites.^72^, ^73^ The decrease in the number of virions caused by HBIG should decrease the viral substrate for antivirals, thus reducing drug‐resistant mutants' emergence.^37^ Thanks to all these mechanisms, HBIG presents a well‐known anti‐inflammatory effect, which could have a substantial impact not only on HBV relapse but also on the overall post‐LT survival rates.

The principal limitation of such an approach is that combination therapy is more expensive than monotherapy with either agent alone.^6^ Moreover, several other unresolved issues should be considered in the use of HBIG, such as their duration (definite vs indefinite), dose (low vs high) and route of administration (intravenous, intramuscular or subcutaneous).^54^, ^74^, ^75^

Another critical issue to explore is the potential differing effect of low genetic barrier‐to‐resistance NUC (LAM) compared to the more recently introduced high genetic barrier‐to‐resistance NUC (ADV, ETV and TDF). Although we tried to answer this relevant question by analysing the literature data, we could not draw definite conclusions, mainly due to the limited sample size in some sub‐analyses. More in detail, data were sufficient to suggest that the combination of HBIG and NUC is more protective than HBIG alone also if a low genetic barrier‐to‐resistance NUC is used (HBIG+LAM). The small sample size in the sub‐analyses of patients treated with the combination of HBIG and high genetic barrier‐to‐resistance NUC compared to HBIG alone did not allow us to obtain definite results. However, it has to be emphasized that in the sub‐analysis focused on this issue, only 13/319 (4.1%) recurrences were reported in patients treated with HBIG and high genetic barrier‐to‐resistance NUC vs 205/1980 (10.4%) in patients treated with HBIG alone (OR = 0.46; *P* = .31). We feel that the limited sample size was the main limitation for identifying a statistical relevance in this comparison. Indeed, the reported result suggests that the combination HBIG+high genetic barrier‐to‐resistance NUC should be the most efficacious prophylaxis in terms of recurrence rate.^76^, ^77^

Lastly, when HBIG+low genetic barrier‐to‐resistance NUC (LAM) therapy was compared with LAM alone, the combination therapy protective effect was evident. This result can be explained by the synergistic effect of HBIG plus NUC. However, when HBIG+high genetic barrier‐to‐resistance NUC were compared with low or high genetic barrier‐to‐resistance NUC alone, no statistical differences were observed. This observation may have several explanations, such as the small sample size of the tested studies or the presence of potential initial selection biases. However, in the case of HBIG+high genetic barrier‐to‐resistance NUC vs high genetic barrier‐to‐resistance NUC alone, an actual biological effect might be hypothesized to explain the result observed, namely the elevated protective effect offered by high genetic barrier‐to‐resistance NUC alone that might be similar to the one obtained by the combination therapy with HBIG. However, we feel that more studies are needed in this setting, and no definitive answer can be provided based on the currently available evidence.

Some considerations should be made on the limitations of the present meta‐analysis. Firstly, most of the included trials showed low methodological quality, with 42 of the 51 selected studies being retrospective cohorts. The six randomized trials reported included only 214 patients altogether, namely 3.4% only of the entire selected population.

Secondly, most nonrandomized studies compared an earlier period in which HBIG monotherapy was used to a later period in which combination therapy or NUC alone therapy was introduced. Consequently, potential confounding variables might not be equally distributed in between study arms, including demographic variables, co‐infection with other viruses (ie HDV and HCV), acute vs chronic HBV‐related liver disease, type of immunosuppression used after LT, pre‐LT therapy with NUC, HBV DNA status before LT, presence of mutations, resistance to LAM and the presence of HCC before transplantation. Moreover, it might be expected that HBV recurrence rates are lower in the most recent cohorts due to the improved management of patients and a better knowledge of the disease, therefore influencing the observed results.

Another relevant time‐dependent change observed is connected with using the most recent high genetic barrier‐to‐resistance NUC with respect to the LAM. LAM is no more considered a prophylactic drug used in HBV patients undergoing a LT. Therefore, the results obtained in this meta‐analysis should be considered in light of the fact that also LAM‐related studies were considered.

Lastly, although HBV recurrence still represents an important issue after LT, it looks not to have the same prognostic significance as in former transplant periods. Overall, post‐LT survival rather than HBV relapse should represent the most critical outcome variable since HBV recurrence may usually be treated appropriately nowadays. Unfortunately, the possibility of constructing a meta‐analysis aimed at using graft or patient survival instead of HBV recurrence is limited by the scarce information reported in the papers. HBV relapse still represents the main goal in the great majority of the studies focused on this issue.

Considering these aspects, we can only partially suggest some recommendations on the best practice to adopt for post‐LT HBV prophylactic management. We can hypothesize that using combination therapy is superior to HBIG alone, and this hypothesis also holds when a low genetic barrier‐to‐resistance NUC is used. Moreover, the combination of HBIG+NUC appears to be superior also over NUC alone. However, HBIG alone appears to be superior when compared with low genetic barrier‐to‐resistance NUC alone. No definite conclusions can be drawn in comparing high genetic barrier‐to‐resistance NUC vs HBIG alone due to the small sample size of the studies evaluated, where two patients had recurrence versus none. Unfortunately, the present meta‐analysis could not definitively clarify the effect of high genetic barrier‐to‐resistance NUC compared with all the other combinations. We also did not explore the practical impact of indefinite vs definite use of HBIG, despite a meta‐analysis recently published in 2020 showed that a finite combination of HBIG and NUC should represent a valid alternative to lifelong dual therapy.^71^

In conclusion, the prophylactic role of HBIG is relevant in preventing HBV recurrence after transplantation. Its combination with NUC gives the best results in terms of protection against the risk of recurrence. The present results should be considered in light of the fact that also old studies based on the prophylactic use of lamivudine were considered. More studies exploring the role of high genetic barrier‐to‐resistance NUC and the impact of protocols with definite use of HBIG are needed.

## CONFLICT OF INTEREST

The authors have no conflict of interest to declare.

## AUTHOR CONTRIBUTIONS

QL and EGG contributed to the conception and design of the study; QL and GM contributed to the acquisition of data; QL and GM analysed and interpreted the data; QL drafted the article; MR and EGG critically revised the manuscript; and all authors approved the final version.

## Supporting information

Fig S1

Fig S2

Supplementary Material
