# Supplementary figures and images for: Immunoglobulin, nucleos(t)ide analogues and hepatitis B virus recurrence after liver transplant: A meta‐analysis
Source: Eur J Clin Invest. 2021 May 3;51(8):e13575. doi: 10.1111/eci.13575 (PMC8365701; doi:10.1111/eci.13575)

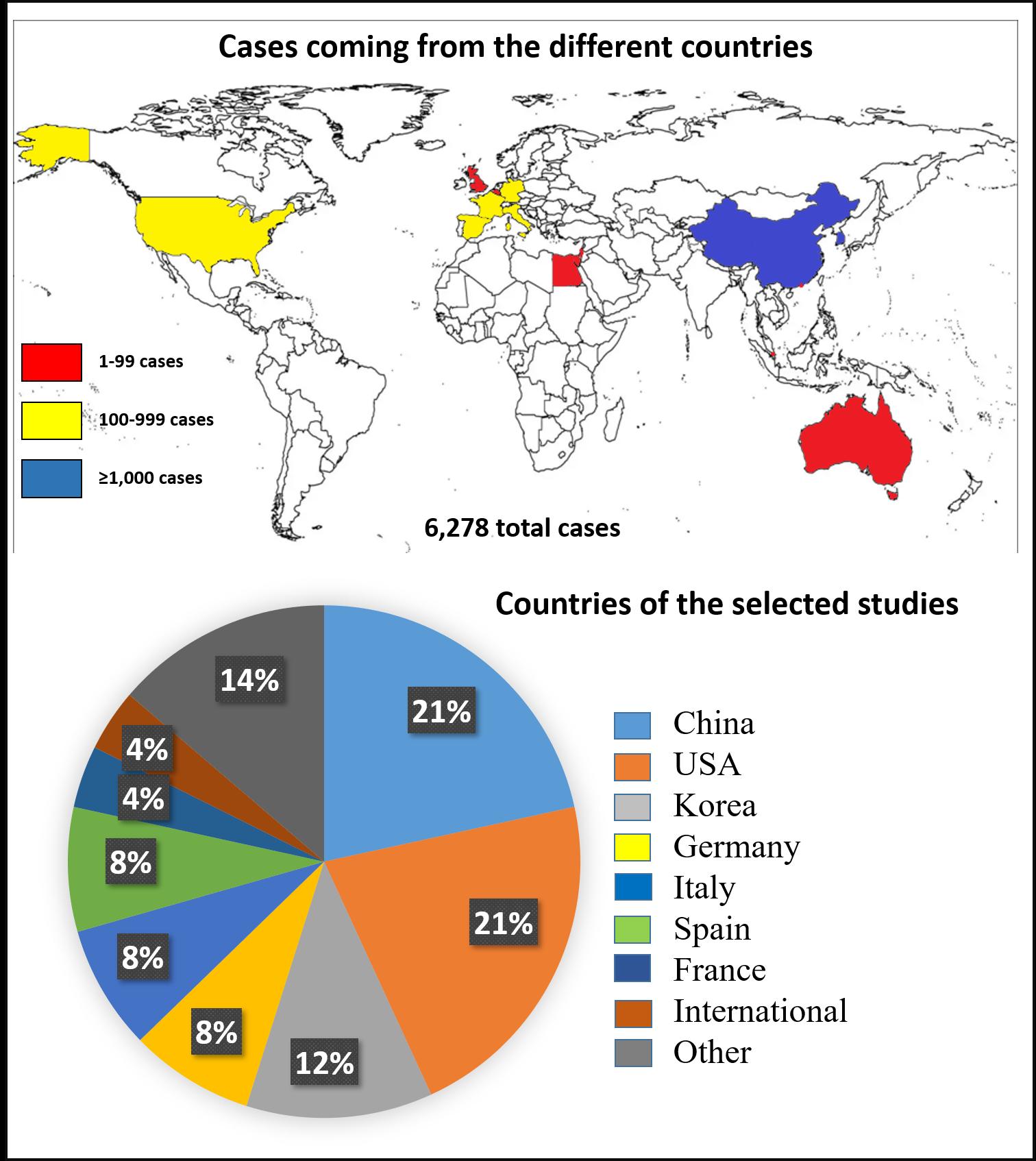

Supplement: Supplementary file 1 — Fig S1 [file ECI-51-e13575-s001.tif]

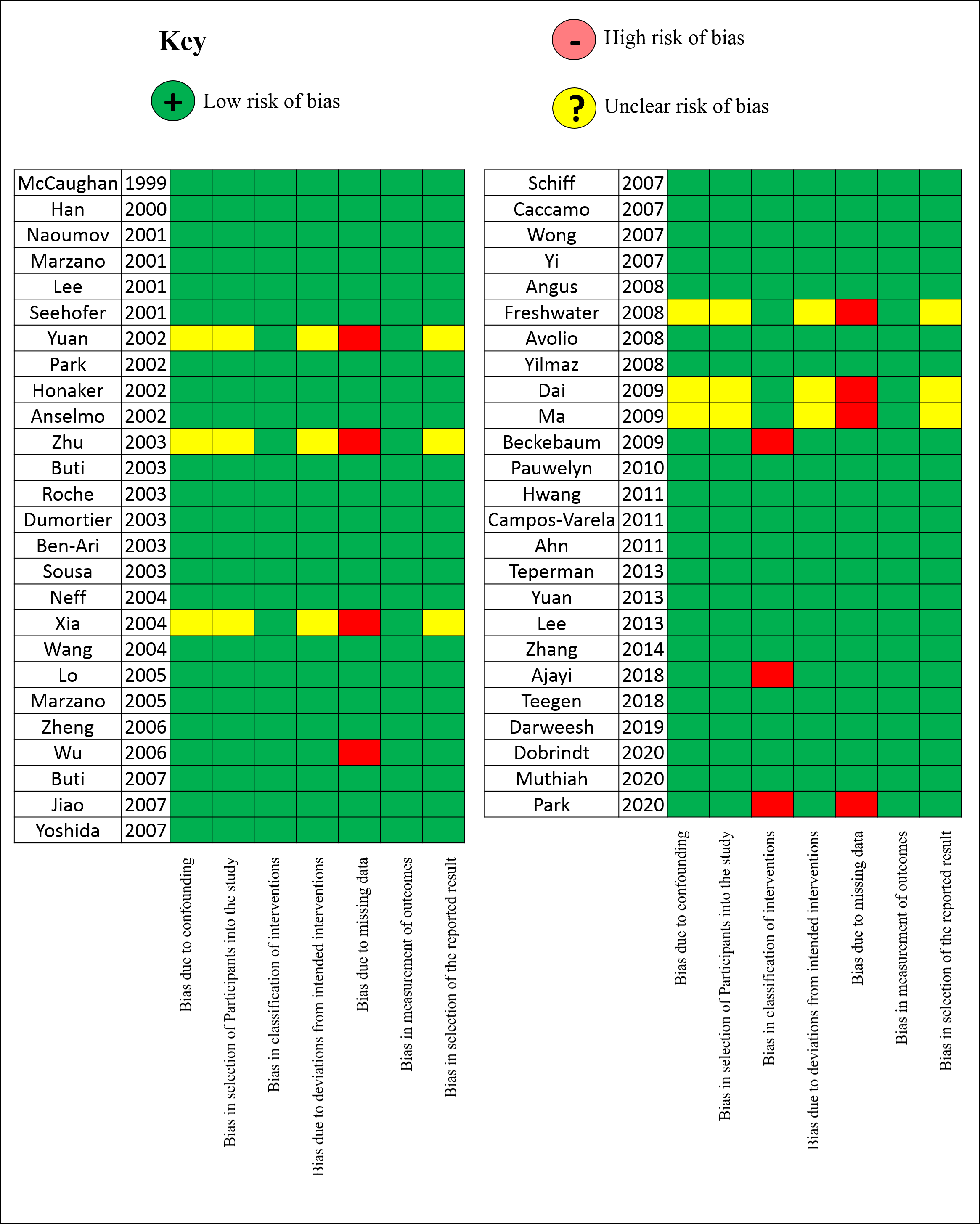

Supplement: Supplementary file 2 — Fig S2 [file ECI-51-e13575-s002.tif]
