# Supplementary material for: Immunoglobulin, nucleos(t)ide analogues and hepatitis B virus recurrence after liver transplant: A meta‐analysis
Source: Eur J Clin Invest. 2021 May 3;51(8):e13575. doi: 10.1111/eci.13575 (PMC8365701; doi:10.1111/eci.13575)
Supplement: Supplementary file 3 — Supplementary Material [file ECI-51-e13575-s003.docx]

**Supplementary Figures**

**Supplementary Figure 1.** Selected articles detailed for geographical distribution.

**Supplementary Figure 2.** Results of the Risk of Bias In Non-randomized Studies of Interventions tool for the extracted articles.
